# Accounting for dominance to improve genomic evaluations of dairy cows for fertility and milk production traits

**DOI:** 10.1186/s12711-016-0186-0

**Published:** 2016-02-01

**Authors:** Hassan Aliloo, Jennie E. Pryce, Oscar González-Recio, Benjamin G. Cocks, Ben J. Hayes

**Affiliations:** Biosciences Research Division, Department of Economic Development, Jobs, Transport and Resources, AgriBio, 5 Ring Road, Bundoora, VIC 3083 Australia; School of Applied Systems Biology, La Trobe University, Bundoora, VIC 3083 Australia; Dairy Futures Cooperative Research Centre (CRC), AgriBio, 5 Ring Road, Bundoora, VIC 3083 Australia; Department of Animal Breeding, INIA, Ctra La Coruña, km 7.5, 28040 Madrid, Spain

## Abstract

**Background:**

Dominance effects may contribute to genetic variation of complex traits in dairy cattle, especially for traits closely related to fitness such as fertility. However, traditional genetic evaluations generally ignore dominance effects and consider additive genetic effects only. Availability of dense single nucleotide polymorphisms (SNPs) panels provides the opportunity to investigate the role of dominance in quantitative variation of complex traits at both the SNP and animal levels. Including dominance effects in the genomic evaluation of animals could also help to increase the accuracy of prediction of future phenotypes. In this study, we estimated additive and dominance variance components for fertility and milk production traits of genotyped Holstein and Jersey cows in Australia. The predictive abilities of a model that accounts for additive effects only (additive), and a model that accounts for both additive and dominance effects (additive + dominance) were compared in a fivefold cross-validation.

**Results:**

Estimates of the proportion of dominance variation relative to phenotypic variation that is captured by SNPs, for production traits, were up to 3.8 and 7.1 % in Holstein and Jersey cows, respectively, whereas, for fertility, they were equal to 1.2 % in Holstein and very close to zero in Jersey cows. We found that including dominance in the model was not consistently advantageous. Based on maximum likelihood ratio tests, the additive + dominance model fitted the data better than the additive model, for milk, fat and protein yields in both breeds. However, regarding the prediction of phenotypes assessed with fivefold cross-validation, including dominance effects in the model improved accuracy only for fat yield in Holstein cows. Regression coefficients of phenotypes on genetic values and mean squared errors of predictions showed that the predictive ability of the additive + dominance model was superior to that of the additive model for some of the traits.

**Conclusions:**

In both breeds, dominance effects were significant (*P* < 0.01) for all milk production traits but not for fertility. Accuracy of prediction of phenotypes was slightly increased by including dominance effects in the genomic evaluation model. Thus, it can help to better identify highly performing individuals and be useful for culling decisions.

## Background

There is limited knowledge on how much genetic variation is explained by non-additive effects in dairy cattle, especially for fertility traits. Dominance is a non-additive genetic effect that arises because of the interactions between alleles at the same locus [1]. The total genetic value of an individual is the sum of additive (i.e. breeding value) and non-additive (e.g. dominance deviation) effects. If the contribution of dominance effects to the total variance is substantial, including dominance effects in genetic evaluation models could improve estimated additive breeding values and lead to better selection decisions. Thus, the prediction of an animal’s future performance would be more accurate, which would help breeders to select animals for replacements or identify animals for culling. Furthermore, taking both dominance and additive effects into account would be useful to select matings and maximize the productive performance of the offspring by exploiting specific combining ability.

However, dominance effects are usually ignored in the genetic evaluation of livestock. In livestock, pedigree information is rarely informative enough to accurately estimate dominance effects since it is difficult to find large populations with considerable dominance relationships such as full-sib families and these effects are often confounded with other non-genetic effects. The prediction of dominance effects using pedigree information is computationally very demanding since it requires large datasets and complex algorithms to compute and invert dominance covariance matrices between individuals [2, 3]. Estimates of dominance variance for various traits in livestock using pedigree information vary considerably between traits and studies, but, in general, have a small to medium (1–34 %) contribution to the total genetic variance [4]. Another reason why dominance effects are generally ignored in the genetic evaluation of animals is that conventional breeding programs aimed at improving the genetic merit of animals rely only on additive gene actions in terms of breeding values, and thus, the prediction of total genetic values has not been considered.

Information from genome-wide single nucleotide polymorphism (SNP) panels have been commonly used in dairy cattle to detect additive effects of SNPs in association studies (e.g. [5]), or to estimate genomic breeding values of animals for selection purposes (e.g. [6, 7]). The availability of large numbers of SNP genotypes also provides an opportunity to study the non-additive gene actions at the individual level. Sun et al. [8] investigated the role of dominance effects for eight traits in Holstein and Jersey breeds using SNP genotypes and showed that small (5–7 %) proportions of the total phenotypic variance could be explained by dominance variance for yield traits. They also reported very small to zero contribution of dominance effects to the variance of non-yield traits in both breeds. In their study, the model that accounted for both additive and dominance effects fitted the data better than the model that accounted for additive effects only for yield traits and resulted in higher prediction accuracies. Ertl et al. [9] estimated variance components for nine milk production and conformation traits in a relatively small population of Bavarian Fleckvieh cows and reported significant dominance variances (28.1–50.5 % of the total genetic variance) for five of the traits studied. Based on simulated data, Da et al. [10] reported that the accuracy of prediction of phenotypes based on total genetic values, defined as the summation of additive and dominance effects, was higher than that of predictions based only on either additive, or dominance effects. In another study using real and simulated data, Nishio and Satoh [11] showed that the contribution of dominance variance to the total phenotypic variance was important and concluded that the accuracy of estimations could be improved by including dominance effects in genetic evaluation models.

The objectives of this study were: (1) to estimate additive and dominance variance components for fertility and milk production traits in Holstein and Jersey cows using covariance matrices between individuals for genomic effects obtained from high-density SNP genotypes; and (2) to investigate whether future phenotypes could be predicted with a higher accuracy by including both additive and dominance effects compared to a model that accounts for additive effects only. We also investigated the relationship between estimated dominance effects and level of inbreeding.

## Methods

### Data

Genotypes were obtained from the Australian national genomic reference population. Genotyping was done using the Illumina BovineSNP50 v2 BeadChip (Illumina, San Diego, CA, USA) and these genotypes were imputed to the 800 k high-density SNP panel using 1785 animals (including 1534 Holstein and 251 Jersey key ancestor bulls and heifers) with genotypes from the Illumina BovineHD BeadChip (Illumina, San Diego, CA, USA). Imputation was done using BEAGLE 3.1 [12] on quality-controlled data as described in [13]. Holstein and Jersey genotypes were imputed together and average squared correlations (r^2^) between imputed genotypes and real genotypes were greater than 0.9 for both breeds. After imputation, 632,003 SNPs distributed over 29 *Bos taurus* autosomes (BTA) and the two sex chromosomes remained. Genotypes of 7902 and 7501 Holstein and 4014 and 3685 Jersey cows with calving interval (CI) and milk production records, respectively, were used in this study.

Phenotypic records were 305-day milk, fat and protein yields and a measure of fertility, i.e. calving interval (CI). Records for all traits were extracted from the Australian Dairy Herd Improvement Scheme (ADHIS; Melbourne, Australia) database. To accurately remove contemporary group effects from the cow phenotypes, a larger dataset comprising 11,294,808 multiple parity records of respectively 3,425,911 and 482,325 Australian Holstein and Jersey lactating cows between 1985 and 2011 were used to estimate fixed effects of age at calving, month of calving, parity and herd-year-season (HYS) management group in a single-trait model. All cows in the full dataset were required to have at least four grandparents of the same breed (Holstein or Jersey). For production traits, milk, fat and protein yields less than 2000 L, 50 and 45 kg and more than 15,000 L, 800 and 600 kg, respectively, were discarded and a minimum and maximum of 270 and 370 days, respectively, for days in milk were set to minimize the effect of extreme lactations on estimates of means and variances. For the fertility dataset, CI had to be within 290 and 550 days to be valid and CI between 551 and 762 days were set to a maximum of 551 days in accordance with [14]. Furthermore, all data were restricted to cows that were between 18 and 36 months old at first calving, that originated from herds with at least 100 lactation records, and for which the sires had at least five daughters in AI programs. More details on the phenotypic records used in this study are in [14] and [15]. The phenotypes of the genotyped animals were corrected for the estimated fixed effects and then, residuals for animals with both phenotypes and genotypes were kept for subsequent statistical analyses. A summary of the phenotypic data on the genotyped animals included in this study is in Table 1. Average and standard deviations of the raw phenotypes for animals with genotypes were similar to those of the population, which confirms that the genotyped animals used in this study represented well the Australian population of Holstein and Jersey cows.

**Table 1:** Summary of the phenotypic information for calving interval (CI), milk, fat and protein yields in genotyped Holstein and Jersey cows

Trait	Holstein		Jersey	
	Nb of records (animals)	Mean ± SD	Nb of records (animals)	Mean ± SD
CI	25,228 (7902)	394 ± 62	11,653 (4014)	397 ± 63
Milk yield	23,283 (7510)	7173 ± 1767	10,170 (3685)	5706 ± 1206
Fat yield	23,283 (7510)	280 ± 67	10,170 (3685)	284 ± 58
Protein yield	23,283 (7510)	237 ± 58	10,170 (3685)	216 ± 46

### Statistical models

Estimates of variance components for each trait were obtained by using the genomic best linear unbiased prediction (GBLUP) method in two univariate models that either included only additive (A) or both additive and dominance (A + D) genetic effects as follows.

For the model with additive effects only (A):

$${\mathbf{y}} = \bf 1_{{\mathbf{n}}} \mu + {\mathbf{Wu}}_{1} + {\mathbf{Wpe}}_{1} + {\mathbf{e}}_{1}.$$

For the model with both additive and dominance effects (A + D):

$${\mathbf{y}} = \bf 1_{{\mathbf{n}}} \mu + {\mathbf{Wu}}_{2} + {\mathbf{Wd}} + {\mathbf{Wpe}}_{2} + {\mathbf{e}}_{2}.$$

**y** is a vector of pre-corrected phenotypes for each trait; **1**_**n**_ is a vector of ones and *µ* is the population mean term; **u**_**1**_ and **u**_**2**_ are vectors of breeding values (BV) of animals and are assumed to be distributed as ${\mathbf{u}}_{1} \sim{\text{N}}\left({0,{\mathbf{G}}\sigma_{{a_{1}}}^{2}} \right)$ and ${\mathbf{u}}_{2} \sim{\text{N}}\left({0,{\mathbf{G}}\sigma_{{a_{2}}}^{2}} \right)$ with **G** being the additive relationship matrix; **d** is the vector of dominance deviations (DV) for animals and is assumed to be distributed as ${\mathbf{d}}\sim{\text{N}}\left({0,{\mathbf{D}}\sigma_{d}^{2}} \right)$ where **D** is the dominance relationship matrix; **pe**_**1**_ and **pe**_**2**_ are vectors of random permanent environmental effects with ${\mathbf{pe}}_{1} \sim{\text{N}}(0,{\mathbf{I}}\sigma_{{pe_{1}}}^{2})$ and ${\mathbf{pe}}_{2} \sim{\text{N}}(0,{\mathbf{I}}\sigma_{{pe_{2}}}^{2})$; **e**_**1**_ and **e**_**2**_ are vectors of random residual terms distributed as ${\mathbf{e}}_{1} \sim{\text{N}}\left({0,{\mathbf{I}}\sigma_{{e_{1}}}^{2}} \right)$ and ${\mathbf{e}}_{2} \sim{\text{N}}\left({0,{\mathbf{I}}\sigma_{{e_{2}}}^{2}} \right)$.** W** is the incidence matrix relating observations to animals and the variances are as follows: $\sigma_{{a_{1}}}^{2}$ and $\sigma_{{a_{2}}}^{2}$ are additive, $\sigma_{{d_{2}}}^{2}$ dominance, $\sigma_{{pe_{2}}}^{2}$ and $\sigma_{{pe_{2}}}^{2}$ permanent environmental, and $\sigma_{{e_{1}}}^{2}$ and $\sigma_{{e_{2}}}^{2}$ residual variances.

The additive (**G**) and dominance (**D**) relationship matrices were constructed based on the information from genome-wide SNPs as described in [10, 16, 17]:

$${\mathbf{G}} = \frac{{{\mathbf{ZZ^{\prime}}}}}{{\sum\nolimits_{i = 1}^{j} {2p_{i} q_{i} } }},$$

$${\mathbf{D}} = \frac{{{\mathbf{MM^{\prime}}}}}{{\sum\nolimits_{i = 1}^{j} {(2p_{i} q_{i} )^{2} } }},$$

where *j* is the total number of SNPs; the elements of** Z** are equal to −2*p*_*i*_, (*q*_*i*_−*p*_*i*_) and 2*q*_*i*_ for aa, Aa and AA genotypes, respectively, with *p*_*i*_ and *q*_*i*_ being the allele frequency of *A* and *a* alleles at marker *i* in the population. For the dominance relationship matrix** D**, aa, Aa and AA genotypes in** M** were respectively coded as −2*p*_*i*_^2^, 2*p*_*i*_*q*_*i*_ and −2*q*_i_^2^. This way of coding the genotypes guarantees the absence of confounding between additive and dominance variances and allows a direct comparison of genomic-based estimates of variance components with the pedigree-based counterparts [17]. Variance components were estimated using ASReml v3.0 [18]. Model A estimates additive breeding values, whereas model A + D provides total genetic values as the sum of the estimated additive breeding values and dominance deviations.

### Inbreeding coefficients

To investigate the relationship between dominance effects and inbreeding, the correlations between estimated dominance deviations and inbreeding coefficients of animals were calculated. Two different measures of inbreeding based on genomic data were used. First, the diagonal elements of the genomic relationship matrix based on [19], which are actually the genomic relationships of an individual with itself relative to a base population, were calculated. These comprise the inbreeding coefficient of each animal plus one, so the inbreeding part was extracted as:

$$G_{F} = \frac{1}{N}\mathop \sum \limits_{i} \frac{{x_{i}^{2} - \left({1 + 2p_{i}} \right)x_{i} + 2p_{i}^{2})}}{{2p_{i} (1 - p_{i})}},$$

where *N* is the total number of SNPs, *p*_*i*_ is the frequency of the most common allele at SNP *i*, and *x*_*i*_ is coded as 0, 1 and 2 for aa, Aa and AA genotypes, respectively. We also calculated the proportion of the SNPs that were homozygous (i.e. the SNP genotypes that were either aa or AA), which was used as another measure of inbreeding.

### Validation

The goodness of fit of models A and A + D were compared based on the maximum likelihood (ML) ratio test. Two times the difference between maximum log of likelihood (LogL) of models A and A + D for each trait were compared to their expected values under a null model with mixed Chi squared distributions with 1 and 0 degree of freedom. In such a comparison, the *χ*_(1)_^2^ at *P* = 0.01 is actually the critical value for the observed test statistic to be in the top 0.005 of the null distribution. To compare the accuracy of prediction of phenotypes obtained from each model, a fivefold cross-validation was performed. Five partitions of the data with approximately equal sizes were generated at random for each trait, except that paternal half-sibs were only present in onefold, so that animals across folds were less related. Such a cross-validation scheme was also used in Khansefid et al. [20] and Bolormaa et al. [21]. Furthermore, partitioning was done such that no predicted cow had daughters in the training dataset, which never occurs in reality. In this setting, no validation animal has paternal half-sibs or descendants in the reference population, which avoids upward bias in the prediction of future phenotypes. Four of the five partitions were combined and used in turn as the reference population (to estimate the additive and dominance effects) and the fifth partition was used as the validation set. Genotypes of validation animals were included in the additive and dominance relationship matrices but their phenotypes were not included in the estimation of genetic values. Correlations, regressions and mean squared errors (MSE) of predictions between the estimated breeding values (additive effects) and total genetic values (additive plus dominance effects) of validation animals by GBLUP and their corrected phenotypes were calculated and averaged for the five validation datasets. Paired two-sample t tests were used to compare correlations and MSE across models A and A + D (following [8]). For regressions, regression coefficients that are closer to 1 are preferred and the differences between these coefficients and 1 were tested by a one-sample *t* test.

To evaluate the effect of accounting for dominance variation on genetic evaluation, animals were ranked based on their estimated breeding values or total genetic values and additional comparisons were made based on Spearman׳s rank correlations across the two models under different hypothetical percentages of selection.

## Results

### Variance components and variance proportions

Estimates of additive variance and additive heritabilities for CI and production traits were similar regardless of whether dominance effects were included in the model (Table 2). Additive heritability estimates and estimates of repeatabilities (additive + dominance + permanent environmental variances divided by phenotypic variance) for milk and protein yields were higher for Jersey than for Holstein cows. For all traits and both breeds, the inclusion of dominance effects in the model did not have a large effect on the estimates of additive and residual components, but it reduced the permanent environmental variances, which indicates that the permanent environmental effects appear to capture the main part of the dominance variance when the model does not specifically account for dominance. Therefore, the sums of the additive, dominance and permanent environmental variance components were similar between the two models in all analyses. Dominance variance explained up to 3.8 and 7.1 % of the total phenotypic variance of production traits for Holstein and Jersey cows, respectively (Table 2). For CI, dominance accounted for 1.2 % of the variation for Holstein cows, but was very close to zero for Jersey cows.

**Table 2:** Variance components, proportion of variances over total phenotypic variance and heritabilities for calving interval (CI), milk, fat and protein yields in Holstein and Jersey cows

Breed	Model	Parameter	Trait			
			CI	Milk yield	Fat yield	Protein yield
Holstein	A	*σ*_*a*_^2^	58 (12)	239,580 (15,500)	257 (20)	145 (12)
		*σ*_*perm*_^2^	43 (18)	150,320 (10,569)	255 (16)	171 (10)
		*σ*_*e*_^2^	2681 (28)	546,590 (6487)	889 (11)	522 (6)
		*h*_*a*_^2^	0.021 (0.004)	0.256 (0.014)	0.184 (0.013)	0.174 (0.013)
		(*σ*_*a*_^2^ + *σ*_*perm*_^2^)/*σ*_*p*_^2^	0.036 (0.006)	0.416 (0.009)	0.366 (0.009)	0.378 (0.009)
	A + D	*σ*_*a*_^2^	58 (12)	239,810 (15,486)	257 (20)	145 (12)
		*σ*_*d*_^2^	34 (22)	30,492 (12,464)	54 (20)	27 (12)
		*σ*_*perm*_^2^	8 (29)	118,360 (16,161)	200 (25)	143 (15)
		*σ*_*e*_^2^	2680 (28)	546,520 (6486)	889 (11)	522 (6)
		*h*_*a*_^2^	0.021 (0.004)	0.256 (0.014)	0.184 (0.013)	0.174 (0.013)
		*h*_*d*_^2^	0.012 (0.008)	0.033 (0.013)	0.038 (0.014)	0.032 (0.014)
		*σ*_*d*_^2^/(*σ*_*a*_^2^ + *σ*_*d*_^2^)	0.366	0.113	0.173	0.155
		(*σ*_*a*_^2^ + *σ*_*d*_^2^ + *σ*_*perm*_^2^)/*σ*_*p*_^2^	0.036 (0.006)	0.416 (0.009)	0.365 (0.009)	0.377 (0.009)
Jersey	A	*σ*_*a*_^2^	63 (20)	137,410 (11,868)	189 (21)	106 (11)
		*σ*_*perm*_^2^	114 (31)	98,832 (7852)	223 (17)	131 (9)
		*σ*_*e*_^2^	2749 (43)	214,400 (3720)	606 (10)	281 (5)
		*h*_*a*_^2^	0.022 (0.007)	0.305 (0.022)	0.186 (0.019)	0.204 (0.020)
		(*σ*_*a*_^2^ + *σ*_*perm*_^2^)/*σ*_*p*_^2^	0.061 (0.009)	0.524 (0.011)	0.405 (0.012)	0.457 (0.012)
	A + D	*σ*_*a*_^2^	63 (20)	137,800 (11,858)	189 (21)	105 (11)
		*σ*_*d*_^2^	0	23,089 (8892)	67 (21)	37 (11)
		*σ*_*perm*_^2^	114 (31)	74,510 (11,473)	153 (26)	93 (14)
		*σ*_*e*_^2^	2749 (43)	214,450 (3721)	606 (10)	281 (5)
		*h*_*a*_^2^	0.022 (0.007)	0.306 (0.022)	0.186 (0.019)	0.204 (0.020)
		*h*_*d*_^2^	0	0.051 (0.020)	0.066 (0.020)	0.071 (0.021)
		*σ*_*d*_^2^/(*σ*_*a*_^2^ + *σ*_*d*_^2^)	0	0.144	0.263	0.258
		(*σ*_*a*_^2^ + *σ*_*d*_^2^ + *σ*_*perm*_^2^)/*σ*_*p*_^2^	0.061 (0.009)	0.523 (0.011)	0.403 (0.012)	0.455 (0.012)

Model A = taking only additive effects into account

Model A + D = taking both additive and dominance effects into account

*σ*_*a*_^2^ = additive variance

*σ*_*perm*_^2^ = permanent environmental variance

*σ*_*e*_^2^ = residual variance

*h*_*a*_^2^ = additive heritability

*σ*_*p*_^2^ = total phenotypic variance

*σ*_*d*_^2^ = dominance variance

*h*_*d*_^2^ = dominance heritability

Standard errors (SE) in parentheses

### Relationship between dominance and inbreeding

Correlations between dominance deviations obtained from the dominance model and two measures of inbreeding level (proportion of homozygous SNPs and inbreeding coefficient of animals) are in Table 3. Estimated dominance deviations for production traits were negatively correlated and moderate in size with inbreeding level defined as the proportion of homozygous SNPs, which indicates that animals with larger positive dominance effects were less inbred. Correlations of dominance deviations with animal inbreeding coefficients were also negative but stronger than those with proportion of homozygous SNPs. For CI in Holstein cows, negative dominance deviations, i.e. favourable dominance effects for CI, were associated with lower values of both measures of animal inbreeding.

**Table 3:** Correlations between estimated dominance deviations from model A + D and inbreeding measures for calving interval (CI), milk, fat and protein yields in Holstein and Jersey cows

Breed	Trait	Inbreeding measures	
		Proportion of homozygous SNPs	Inbreeding coefficient
Holstein	CI	0.091	0.127
	Milk yield	−0.210	−0.317
	Fat yield	−0.208	−0.308
	Protein yield	−0.218	−0.321
Jersey	CI	0^a^	0^a^
	Milk yield	−0.225	−0.311
	Fat yield	−0.249	−0.355
	Protein yield	−0.248	−0.345

Model A + D = taking both additive and dominance effects into account

^a^Since estimated dominance deviations for CI in Jersey cows were very close to zero, the estimated correlations were also very close to zero

### Comparison of models

#### Goodness of fit

The maximum LogL values from models A and A + D and the *P* values of their conservative likelihood ratio tests are in Table 4. For all traits and both breeds except for CI in Jersey cows, the value of the maximum LogL was greater with model A + D than with model A, which indicates that model A + D fits the data better. The differences between the highest LogL values with the two models were significant (*P* < 0.01) for all production traits but not for CI, which shows less dominance variation than for production traits in the Holstein breed and no dominance variance in the Jersey breed.

**Table 4:** Comparison of the goodness of fit of models A and A + D based on conservative maximum likelihood (ML) ratio test for calving interval (CI), milk, fat and protein yields in Holstein and Jersey cows

Breed	Parameter	Model	Trait			
			CI	Milk yield	Fat yield	Protein yield
Holstein	Log of ML	A	−112,604	−154,634	−85,995	−80,469
		A + D	−112,603	−154,629	−85,990	−80,465
	*P* value*		0.0569	0.00095	0.00095	0.0026
Jersey	Log of ML	A	−52,292	−69,655	−39,350	−35,684
		A + D	−52,292	−69,650	−39,343	−35,676
	*P* value*		–	0.0012	5.91E-05	3.23E-05

Model A = taking only additive effects into account

Model A + D = taking both additive and dominance effects into account

* *P* values of the Chi square tests calculated as *χ* ^2^ = −2(log ML_A_ − log ML_A + D_) with 1 degree of freedom from a mixture of Chi squared distributions with 1 and 0 degree freedom

#### Prediction accuracy

We assessed the predictive ability of the models in three ways: (1) Pearson’s correlations between estimated genetic values and phenotypes adjusted for fixed effects; (2) regression coefficients of adjusted phenotypes on estimated genetic values; and (3) mean squared errors (MSE) of prediction of adjusted phenotypes from the estimated genetic values. The results are presented as averages for five validation datasets.

Average correlations between estimated breeding values or total genetic values and adjusted phenotypes in the validation data for fivefold cross-validation showed a slightly better predictive ability with model A + D than with model A for most of the traits (Table 5). Standard errors of these correlations were lower for production traits in the Holstein than in the Jersey breed, which reflects the larger number of observations for prediction of genetic values of Holstein cows. However, the difference between correlations from models A and A + D was statistically significant (*P* < 0.01) only for fat yield in Holstein cows and for the total genetic values compared to predictions based on additive effects only.

**Table 5:** Accuracy of prediction of phenotypes in cross-validation

Breed	Model	Parameter	Trait			
			CI	Milk yield	Fat yield	Protein yield
Holstein	A	BV	0.080 (0.008)	0.346 (0.011)	0.270 (0.017)	0.266 (0.018)
	A + D	BV	0.080 (0.008)	0.347 (0.011)	0.270 (0.017)	0.267 (0.018)
		DV	0.025 (0.014)	0.038 (0.014)	0.038 (0.003)	0.035 (0.008)
		BV + DV	0.082 (0.011)	0.348 (0.011)	0.273 (0.017)	0.269 (0.018)
	A vs. A + D	*P* value (BV_A_ vs. BV_A + D_)	0.9476	0.0169	0.4517	0.2360
		*P* value (BV_A_ vs. [BV + DV]_A + D_)	0.5456	0.0694	0.0060	0.0202
Jersey	A	BV	0.094 (0.007)	0.368 (0.017)	0.243 (0.025)	0.256 (0.025)
	A + D	BV	0.094 (0.007)	0.367 (0.018)	0.244 (0.026)	0.255 (0.026)
		DV	0.005 (0.014)	0.046 (0.006)	0.059 (0.018)	0.071 (0.015)
		BV + DV	0.094 (0.007)	0.370 (0.017)	0.250 (0.024)	0.262 (0.023)
	A vs. A + D	*P* value (BV_A_ vs. BV_A + D_)	0.6069	0.8485	0.7820	0.6068
		*P* value (BV_A_ vs. [BV + DV]_A + D_)	0.6000	0.1275	0.1355	0.1727

Average correlations between estimated genetic effects (breeding values [BV], dominance deviations [DV] or total genetic values [BV + DV]) obtained from models A (additive effects only) and A + D (additive and dominance effects) and adjusted phenotypes for calving interval (CI), milk, fat and protein yield in Holstein and Jersey cows based on fivefold cross validation

Standard errors (SE) in parentheses

Average regression coefficients of adjusted phenotypes on estimated genetic values obtained with models A (*b*_adjusted phenotype, BV_) and A + D (*b*_adjusted phenotype, BV + DV_) in the fivefold cross-validation analysis are in Table 6. The estimated regression coefficients for model A + D were closer to 1 than those for model A in all cases except for CI in Holstein and milk yield in Jersey cows but these were not significantly different from 1 for all traits and both breeds.

**Table 6:** Regression coefficients of models A and A + D in cross-validation

Breed	Model	Parameter	Trait			
			CI	Milk yield	Fat yield	Protein yield
Holstein	A	BV	1.090 (0.187)	0.985 (0.051)	0.995 (0.080)	0.983 (0.094)
	A + D	BV + DV	1.104 (0.183)	0.987 (0.049)	0.999 (0.082)	0.986 (0.093)
	A vs. A + D	*P* value (BV_A_ vs. 1)	0.6563	0.7800	0.9488	0.8686
		*P* value ([BV + DV]_A + D_ vs. 1)	0.5986	0.8080	0.9892	0.8879
Jersey	A	BV	1.637 (0.178)	1.011 (0.093)	0.911 (0.133)	0.925 (0.120)
	A + D	BV + DV	1.629 (0.176)	1.012 (0.095)	0.917 (0.143)	0.929 (0.130)
	A vs. A + D	*P* value (BV_A_ vs. 1)	0.02	0.91	0.54	0.56
		*P* value ([BV + DV]_A + D_ vs. 1)	0.02	0.90	0.59	0.61

Average regression coefficients between estimated genetic effects (breeding values [BV] or total genetic values [BV + DV]) obtained from models A (additive effects only) and A + D (additive and dominance effects) and adjusted phenotypes for calving interval (CI), milk, fat and protein yield in Holstein and Jersey cows based on fivefold cross validation

Standard errors (SE) in parentheses

Mean squared errors of predictions also showed a better predictive ability with model A + D than with model A (Table 7), i.e. smaller values were always found with model A + D than with model A. Nevertheless, the difference between models was only statistically significant (*P* < 0.01) for fat and protein yields in Holstein cows (Table 7).

**Table 7:** Mean squared error of predictions from models A and A + D in cross-validation

Breed	Model	Parameter	Trait			
			CI	Milk yield	Fat yield	Protein yield
Holstein	A	BV	1451.26 (45.33)	589,029.83 (10,194.11)	883.24 (11.72)	548.16 (12.58)
	A + D	BV + DV	1450.84 (44.97)	587,372.70 (10,098.80)	880.95 (11.85)	546.86 (12.47)
	A vs. A + D	*P* value (BV_A_ vs. [BV + DV]_A + D_)	0.5505	0.0122	0.0056	0.0007
Jersey	A	BV	1847.03 (50.92)	302,351.20 (11,723.35)	684.67 (26.29)	361.06 (14.41)
	A + D	BV + DV	1847.03 (50.92)	301,162.79 (11,690.03)	679.29 (25.51)	358.46 (14.57)
	A vs. A + D	*P* value (BV_A_ vs. [BV + DV]_A + D_)	0.2835	0.0174	0.0281	0.0296

Average mean squared errors (MSE) between estimated genetic effects (breeding values [BV] or total genetic values [BV + DV]) obtained from models A (additive effects only) and A + D (additive and dominance effects) and adjusted phenotypes for calving interval (CI), milk, fat and protein yield in Holstein and Jersey cows based on fivefold cross validation

Standard errors (SE) in parentheses

#### Effect of dominance on the evaluation of animals

When animals were ranked using only their estimated breeding values obtained from models A or A + D, Spearman’s rank correlations were always greater than 0.9 for all traits (results are not shown). The average rank correlations of animals in the five validation sets according to their estimated breeding values from model A and total genetic values from model A + D are in Table 8. Correlations between the ranks of animals based on total genetic values were lower which indicated a slight re-ranking of animals especially for the top ranking animals.

**Table 8:** Rank correlations according to breeding values (model A) and total genetic values (model A + D)

Percentage (%)	CI	Milk yield	Fat yield	Protein yield
	Holstein			
10	0.846 (0.111)	0.949 (0.037)	0.886 (0.036)	0.934 (0.032)
25	0.894 (0.066)	0.966 (0.024)	0.927 (0.029)	0.949 (0.027)
50	0.928 (0.045)	0.980 (0.014)	0.954 (0.021)	0.971 (0.016)
75	0.959 (0.026)	0.988 (0.009)	0.974 (0.012)	0.983 (0.009)
100	0.980 (0.013)	0.994 (0.004)	0.988 (0.006)	0.992 (0.005)
	Jersey			
10	1 (0)	0.885 (0.037)	0.734 (0.088)	0.676 (0.110)
25	1 (0)	0.918 (0.024)	0.824 (0.065)	0.824 (0.054)
50	1 (0)	0.954 (0.008)	0.877 (0.059)	0.872 (0.040)
75	1 (0)	0.975 (0.004)	0.921 (0.041)	0.923 (0.027)
100	1 (0)	0.988 (0.002)	0.962 (0.019)	0.963 (0.013)

Average rank correlations based on estimated breeding values (BV) from model A (additive effects only) and total genetic values (BV + DV) from model A + D (additive and dominance effects) for calving interval (CI), milk, fat and protein yield in Holstein and Jersey cows based on fivefold cross validation across different percentages of selection

Standard errors (SE) in parentheses

## Discussion

The proportion of dominance variance relative to phenotypic variance was small for all traits studied here. Dominance variance accounted for 3.3, 3.8 and 3.2 % of the phenotypic variance for milk, fat and protein yields, respectively in Holstein cows. Larger proportions of dominance variance relative to phenotypic variance were estimated for milk (5.1 %), fat (6.6 %) and protein (7.1 %) yields in Jersey cows. For CI, estimated dominance variance was about 1.2 % in Holstein but very close to zero in Jersey cows. The additive and dominance effects estimated for a SNP depend on the extent of linkage disequilibrium (LD) between the SNP and the causative mutation. Specifically, the proportion of additive genetic variance observed at the SNP depends on the r^2^ (squared coefficient of correlation between SNP and causative mutation), whereas for the dominance variance, it depends on (r^2^)^2^, i.e. r^4^ [22]. Thus, more data is required for the accurate estimation of dominance effects and variances than for that of additive effects. In addition, larger datasets are necessary to efficiently differentiate permanent environmental effects from the dominance variance otherwise the dominance variance may be substantially over-estimated due to the inclusion of variance from permanent environment effects. The size of the dataset on CI records was smaller for Jersey than for Holstein cows in our study, which might be the reason of the disparity that was observed in estimates of dominance variance for fertility between the two breeds. When the magnitude of dominance variance was compared to the total genetic variance of traits, the contribution of dominance variance was important, such that it explained up to 37 and 17 % of the total genetic variance for CI and yield traits, respectively in Holstein cows. The ratio of dominance variance relative to total genetic variance was up to 26 % for yield traits in Jersey cows.

Estimation of additive and non-additive variances for economically important traits in dairy cattle is usually based on pedigree relationship matrices. Tempelman and Burnside [23] reported considerable dominance variance for fat yield (24 %) in the Canadian Holstein breed, while Miglior et al. [24] estimated a rather small dominance variance (up to 3 %) for milk production traits in Holstein cows in Canada. Van Tassell et al. [25] reported dominance variances of up to 5 % for yield traits in Holstein cows in the United States and Fuerst and Sölkner [4] concluded that dominance variance was important for all milk production traits in Austrian dairy cattle. Overall, these results are in agreement with our findings for dominance variance of milk production traits in genotyped Holstein (3.8 %) and Jersey (7.1 %) cows in Australia. For fertility, Hoeschele [26] showed that the dominance variance can be as large as, or even larger than, the additive variance in US Holstein cows, and Druet et al. [27] reported similar values for additive and dominance variances for fertility traits in Austrian Simmental and Brown Swiss dairy cattle. However, we did not find large dominance variances for Holstein (1.2 %) or Jersey (close to zero) cows in this study.

The use of genomic data instead of pedigree information in genetic evaluations has led to a renewed interest in the prediction of non-additive genetic effects. Sun et al. [8] reported dominance heritabilities of up to 5 and 7 % for milk production traits in genotyped Holstein and Jersey cows, respectively, in the United States, which are similar to the values obtained in our study. Conversely, Ertl et al. [9] found much larger proportions of dominance variance relative to genetic and phenotypic variances for milk (up to 26.4 % dominance heritability) and conformation traits (up to 11.7 % dominance heritability) in genotyped Fleckvieh cows in Germany. However, their estimates may have been biased upwards because they did not include permanent environmental effects for repeated records in their model based on the relatively small dataset that they analysed. Our results showed that accurate estimation of dominance variance for repeated measurements required fitting permanent environmental effects in the model, since it absorbed most of the dominance variance when dominance effects were not accounted for. In another study, Wittenburg et al. [28] analysed 17 milk performance (yield and component) traits on 1295 Holstein cows in Germany and found that most of the traits under investigation were mainly affected by additive genetic effects, but protein (*h*_*d*_^2^ = 0.23) and casein contents (*h*_*d*_^2^ = 0.21) showed a significant (*P* < 0.05) contribution of dominance variance to the total phenotypic variance.

The range of the estimated contributions of non-additive genetic variance to total genetic variance varies considerably across studies. The reasons that may explain these inconsistencies could be differences in the definition of traits, the size of the dataset used in the analyses, inconsistencies in the estimation of dominance relationships between individuals (pedigree vs. genomic data), the models used to estimate yield deviations, pre-selection of genotyped animals, breed- and population-specific differences and also confounding between additive and dominance variances as argued by [17]. For example, Sun et al. [8] showed that using different methods to construct the dominance relationship matrix results in different estimates of dominance heritabilities for the same set of genotyped animals.

Variation in the level of inbreeding within populations or breeds could be another important factor to explain the inconsistencies reported for the level of contribution of dominance gene actions to the total phenotypic variation of the trait of interest. Dominance variance increases with inbreeding up to a point (inbreeding level of about 0.25 [29]) and populations with higher levels of inbreeding have a larger proportion of the total phenotypic variation arising from dominance effects [1]. Consistent with this, we observed that dominance heritability was higher for yield traits in Jersey than in Holstein cows. Accordingly, Jersey cows have higher average levels of inbreeding than Holstein cows in Australia [30]. These results are in agreement with the findings of Sun et al. [8] who also reported a larger contribution of the dominance variance to total variance for milk production traits in Jersey compared to Holstein cattle in the US. Misztal et al. [3] investigated the relationship between dominance variance and inbreeding using pedigree information for linear traits in US Holstein cattle and found that greater inbreeding depression was associated with higher estimates of the dominance variance.

Inbreeding influences complex traits by increasing the number of homozygous loci and corresponding reductions in heterozygosity. It has been hypothesised that the action of (semi) lethal recessive mutations is the major reason for this [31], but it could also be due to the loss of dominant gene actions through a reduced number of heterozygous loci. In our study, we found that the estimated dominance deviations had unfavourable correlations (in the direction of poor fertility and less production) with the percentage of homozygous SNPs and also with inbreeding coefficients. In other words, animals with higher inbreeding levels gained less from dominance effects in terms of improved performance. It should be noted that this is different from inbreeding at the population level where even small dominance effects can cause a sizable difference in the phenotypes of individuals and can be a significant source of variation in inbred populations. Pryce et al. [30] investigated inbreeding depression effects in Australian Holstein and Jersey cows and showed that the increase in inbreeding was associated with a decrease in phenotypic means of fertility and milk production traits. Dominance effects could counter this effect by helping to identify animals with superior performance for characters of economic importance.

Using additive plus dominance effects, rather than additive effects only, led to slightly better predictions of phenotypes (Table 5). The accuracy of predicting missing phenotypes based on total genetic values was higher for milk production traits than for CI, probably because of greater additive and dominance heritabilities of production traits. However, these accuracies were not significantly different between models A and A + D except for fat yield (*P* < 0.01) in Holstein cows. We found similar results to those reported in [8], which showed that model A + D performed better than model A in terms of predicting phenotypes for production traits. These authors had access to a larger number of genotyped animals compared to our study and, thus, the size of their genomic reference population was larger, and they had more power in their analysis, which may explain why they observed larger differences between models. However, they did not find significant differences between models A and A + D for fertility and somatic cell score. Another possible explanation could be the small proportion of full-sibs and small dominance relationships between the training and validation datasets [9]. Regression coefficients (Table 6) and MSE (Table 7) also favoured model A + D over model A. Average regression coefficients of adjusted phenotypes on estimated genetic values with model A + D were closer to 1 for most traits than those with model A, which shows a better predictive ability for model A + D. Besides, MSE of predictions were smaller with model A + D than with model A which further supports the advantage of model A + D. These results indicate that the goodness of fit and consequently the accuracy of genomic predictions can be improved by including dominance effects in models for predicting the future performance of animals.

One of the important consequences of ignoring non-additive effects could be the biased evaluation of individuals and subsequently incorrect rankings based on estimated breeding values. It has been argued in other studies that including dominance effects in genetic evaluation models could result in more accurate estimates of additive genetic variance and breeding values, thereby increasing the accuracy of predictions [32, 33]. This also provides another valuable criterion, dominance deviation, which can be incorporated in culling and mating decisions. The future performance of an animal is a function of both additive genetic merit (i.e., estimated breeding value) and non-additive genetic merit (i.e. dominance merit and epistasis merit). Culling decisions can be based on total genetic values instead of breeding values only to identify the most profitable cows. In our study, when animals were ranked based on breeding values plus dominance deviations, rankings slightly differed from those based on breeding values only (e.g. rank correlations of 0.9 in the top 75 % Jersey individuals for fat and protein yields) which reflects some re-ranking, especially among the top animals. This is important because animals with higher values of additive plus dominance effects can be selected as replacements in preference to animals with only high additive breeding values; this strategy is expected to improve the overall performance of the herd.

Another use of dominance information would be to allocate matings by considering specific combining ability of the parents and predicting the outcome of every possible mating to choose the matings that can maximize the productive performance of the offspring. Ertl et al. [9] compared the outcome of two different scenarios i.e. selection based on breeding values versus selection based on total genetic values in Fleckvieh cows and showed that the latter resulted in progeny with a greater expected total genetic superiority, i.e. 14.8 % for milk yield and 27.8 % for protein yield and that it reduced the expected additive genetic gain by only 4.5 % for milk yield and 2.6 % for protein yield. Sun et al. [34] investigated the performance of mating programs with and without dominance effects for maximizing the expected progeny value (EPV) for US Holstein and Jersey milk yields and showed that regardless of the sire selection method, mate allocation algorithm and pedigree or genomic inbreeding source, inclusion of dominance effects in addition to additive effects, always resulted in higher EPV for milk yield. Exploiting non-additive sources of genetic variation is likely to become increasingly important as the dairy sector becomes more competitive and breeding goals become more complex.

The small dominance variances estimated for CI could also be related to the measure of fertility used in this study. Calving interval has a low heritability, and the very large environmental variance makes it difficult to estimate dominance effects (and additive effects for that matter) accurately. Other fertility measures with a higher heritability including detailed traits may help to better understand the role of non-additive gene actions for traits that are closely related with fitness. For example, Bolormaa et al. [21] estimated a relatively large genomic dominance variance (18 % of the total phenotypic variance) for age at first detected corpus luteum in beef cattle.

## Conclusions

Dominance variance contributed up to 3.8 and 7.1 % of the phenotypic variation of milk production traits in Australian Holstein and Jersey cows, respectively, whereas this contribution was much smaller in the case of CI. However, when compared to the additive component, dominance represented a significant proportion of the total genetic variance for all milk production traits. Dominance correlated unfavourably with inbreeding which suggests that more inbred animals benefit less from dominance in the direction of improved performance. The better performance (better goodness of fit and prediction accuracy) of model A + D for some traits compared to model A suggests that dominance should be included in models for the genetic evaluation of animals to improve the accuracy of prediction of future phenotypes. This could also become a useful tool for culling decisions on farms, and use of the total genetic of potential progeny in mating plans may improve progeny performance.
